# Association of Heart Rate Variability and Acceleration Plethysmography with Systemic Comorbidity Burden in Patients with Glaucoma

**DOI:** 10.3390/biomedicines13092155

**Published:** 2025-09-04

**Authors:** Yuto Yoshida, Hinako Takei, Misaki Ukisu, Keigo Takagi, Masaki Tanito

**Affiliations:** Department of Ophthalmology, Shimane University Faculty of Medicine, Izumo 693-8501, Japan; y-yoshida@juntendo.ac.jp (Y.Y.); kei918.oor@icloud.com (K.T.)

**Keywords:** heart rate variability, autonomic nervous system, accelerated plethysmography, primary open angle glaucoma, exfoliation glaucoma, age-adjusted Charlson Comorbidity Index

## Abstract

**Background**: Autonomic nervous system (ANS) and vascular factors are associated with glaucoma. However, the association between systemic comorbidity burden and ANS and hemodynamic function in patients with glaucoma remains unclear. This study aimed to examine the association between heart rate variability (HRV) and acceleration plethysmography (APG) parameters and the age-adjusted Charlson Comorbidity Index (ACCI) in patients with glaucoma. **Methods**: A total of 260 subjects (260 eyes), including 186 with primary open-angle glaucoma (PG) and 74 with exfoliation glaucoma (EG), were enrolled at Shimane University Hospital from June 2023 to July 2024. HRV and APG were assessed using a sphygmograph (TAS9 Pulse Analyzer Plus View). HRV parameters included time-domain measures (SDNN, RMSSD, CVRR) and frequency-domain measures (TP, VLF, LF, HF, LF/HF). APG parameters included the a, b, c, d, and e components of the accelerated pulse wave, and the following vascular types: Type A, Type B, and Type C. The association between ACCI and HRV and APG parameters was evaluated using Spearman’s rank correlation and multivariate regression adjusted for sex, body mass index, pulse rate, systolic and diastolic blood pressure, intraocular pressure, medication score, mean deviation, and glaucoma type. **Results**: By univariate analysis, against ACCI, significant inverse correlations were observed for several parameters: LnLF (R = −0.17, *p* = 0.0062); LnLF/LnHF (R = −0.24, *p* = 0.00012); b peak (R = −0.14, *p* = 0.031); d peak (R = −0.17, *p* = 0.0072); and e peak (R = −0.15, *p* = 0.015). Regarding HRV parameters, multivariate linear regression models showed that ACCI was significantly positively associated with RMSSD (coefficient: 2.861; 95% CI: 0.447 to 5.274) and significantly negatively associated with the frequency-domain parameters LnLF (coefficient: −0.127; 95% CI: −0.245 to −0.009) and LnLF/LnHF (coefficient: −0.038; 95% CI: −0.062 to −0.014). In APG parameters, the c peak was significant associated with ACCI (coefficient: −12.6; 95% CI: −22.5 to −2.69). ACCI was significantly associated with Type B (coefficient: 0.305; 95% CI: 0.057 to 0.552). **Conclusions**: Greater systemic comorbidity burden may be related to impaired ANS regulation and increased vascular stiffness in glaucoma patients.

## 1. Introduction

Globally, glaucoma is one of the leading causes of irreversible blindness [1]. The global number of glaucoma patients is projected to continue rising due to population aging [2]. Increased intraocular pressure (IOP) is the strongest risk factor for glaucoma progression [1,3]. Additionally, vascular factors such as blood pressure (BP) and ocular perfusion pressure (OPP) may contribute to its development and progression [4,5,6,7]. Although the pathogenesis of glaucoma is thought to involve multiple factors, it remains incompletely understood.

Recent evidence suggests that glaucoma may be associated with autonomic nervous system (ANS) dysfunction [8,9,10,11,12], which could contribute to its progression by altering IOP through the regulation of aqueous humor production and outflow [13], and by influencing vascular risk factors such as BP and OPP [13,14,15,16]. The association between heart rate variability (HRV), a marker of autonomic function, and glaucoma has also been demonstrated in a population-based cohort study [17]. In our previous studies, we compared HRV between primary open-angle glaucoma (PG) and exfoliation glaucoma (EG) and reported that sympathetic predominance was significantly greater in EG [11,18].

Moreover, systemic arterial stiffness and atherosclerosis may be associated with the pathogenesis of glaucoma. Several studies reported the association between increased systemic arterial stiffness and glaucoma [19,20,21,22,23]. Additionally, our previous study demonstrated patients with EG may have greater arterial stiffness compared to those with PG or the controls [23].

Despite these findings, the association between autonomic and hemodynamic function and systemic comorbidity burden in patients with glaucoma remains unclear. To address this gap, the present study aimed to examine the association between HRV and APG parameters and the Age-adjusted Charlson Comorbidity Index (ACCI) in patients with glaucoma. Furthermore, we investigated this association separately in patients with PG and those with EG.

## 2. Materials and Methods

### 2.1. Study Design and Participants

Ethics approval was obtained from the Institutional Review Board of Shimane University Hospital (Approval No. 20200228-2; revision dated 27 October 2024). All procedures complied with the Declaration of Helsinki. Informed consent was waived on the basis of public disclosure on the institutional website with an opt-out mechanism. All patient data were anonymized before analysis. Participants with a diagnosis of PG or EG were enrolled at Shimane University from June 2023 to July 2024. Ophthalmologists established the diagnosis of glaucoma using IOP, gonioscopy, optic nerve evaluation via fundus photography and optical coherence tomography (OCT), and visual field assessments. For analysis, one eye per participant was included. If disease was unilateral, we selected the diseased eye. If bilateral, we included the eye with the higher IOP; when equal, the right eye was chosen. Participants with a measured reliability score <95% or with ocular diseases other than PG, EG, or cataract were excluded from the study. Ophthalmic data obtained from patient records included the highest IOP recorded during follow-up, the medication score, and the mean deviation (MD) values derived from the central 30-2 perimetric protocol of the Humphrey Visual Field Analyzer (Carl Zeiss Meditec, Jena, Germany). IOP was measured by Goldmann applanation tonometry (Haag-Streit, Köniz, Switzerland). For the medication score, each topical drug component counted as 1 point, as did each tablet of oral acetazolamide.

### 2.2. Age-Adjusted Charlson Comorbidity Index (ACCI)

The Charlson Comorbidity Index (CCI) quantifies comorbidity burden by assigning weighted scores to 19 chronic conditions, such as cardiovascular, pulmonary, hepatic, renal, and malignant diseases, as well as diabetes and dementia [24]. The total CCI score estimates the 10-year mortality risk, with higher scores reflecting greater disease burden [24]. To enhance its prognostic accuracy in older individuals, ACCI incorporates age by adding 1 point for individuals aged 41–50, 2 points for those 51–60, 3 points for 61–70, 4 points for 71–80, and 5 points for those 81 and older, thereby accounting for both comorbidities and age-related vulnerability [25].

### 2.3. Heart Rate Variability (HRV)

HRV was measured in each participant to assess ANS activity using a sphygmograph device (TAS9 Pulse Analyzer Plus View; YKC Corp., Tokyo, Japan). All measurements were performed during daytime outpatient visits with participants seated, following a standardized 2.5 min protocol at a sampling frequency of 1 kHz. In this study, both time- and frequency-domain HRV parameters were used to assess autonomic function [26,27,28,29,30]. The time-domain parameters included the standard deviation of normal-to-normal intervals (SDNN), reflecting overall autonomic activity; the square root of the mean of the sum of the squared differences between adjacent normal-to-normal intervals (RMSSD), an index of cardiac parasympathetic activity; and the coefficient of variation in R-R intervals (CVRR), indicative of parasympathetic function [26]. The frequency-domain parameters included total power (TP), indicating overall autonomic activity; very-low-frequency (VLF, 0.0033–0.04 Hz), related to thermoregulation and long-term control; low-frequency (LF, 0.04–0.15 Hz), reflecting both sympathetic and parasympathetic activity with baroreflex influence; high-frequency (HF, 0.15–0.40 Hz), representing parasympathetic activity; and the LF/HF ratio, an index of sympathovagal balance, with higher values indicating sympathetic dominance [27]. To approximate a normal distribution for statistical analysis, each parameter was transformed using the natural logarithm and analyzed as LnTP, LnVLF, LnLF, LnHF, and LnLF/LnHF.

### 2.4. Accelerated Plethysmography (APG)

As with HRV parameters, APG parameters were measured using the same device in APG measurement mode. APG measurements were also performed under the same conditions during daytime outpatient visits. The method for calculating APG parameters has been described in detail in our previous study [23]. In this study, the APG parameters included the following five components: a peak, the initial systolic upstroke associated with left ventricular ejection; b peak, the early systolic reflection wave reflecting cardiac output; c peak, the late systolic component reflecting residual blood volume; d peak, the diastolic reflection wave indicating arterial elasticity; e peak, the end-diastolic waveform reflecting peripheral vascular resistance [31,32]. The APG waveform was categorized into the following vascular types: Type A, reflecting elastic and healthy arteries; Type B, reflecting moderate arterial stiffness; Type C, reflecting advanced arterial stiffness.

### 2.5. Statistical Analysis

For all variables, descriptive summaries are provided: continuous data are reported as mean ± standard deviation (SD), and categorical data as number (percentage). Comparisons of PG vs. EG used the independent samples *t*-test for continuous data and the chi-square test for categorical data. Spearman’s rank correlation was performed to examine the correlations between ACCI and HRV and APG parameters. The association between ACCI and HRV and APG parameters was evaluated using multivariate regression, adjusting for sex, body mass index (BMI), pulse rate, systolic and diastolic BP, IOP (highest recorded), medication score, MD, and glaucoma type. In this study, missing data were minimal. No missing values were observed for HRV parameters. For APG parameters, 7 cases (2.7%) had missing values, which were excluded from the corresponding analyses. All statistical analyses were conducted using R (version 4.5.1; R Core Team, Vienna, Austria). A *p*-value of <0.05 was considered indicative of statistical significance.

## 3. Results

This study included 260 subjects (260 eyes): 186 in the PG group and 74 in the EG group. As shown in Table 1, the EG group was significantly older than the PG group (PG: 66.3 ± 12.2 years vs. EG: 74.8 ± 8.53 years, *p* < 0.001). The EG group had a significantly higher ACCI compared to the PG group (PG: 3.25 ± 1.51 vs. EG: 4.00 ± 1.21, *p* < 0.001). The IOP was significantly higher in the EG group compared with the PG group (PG: 20.3 ± 7.39 mmHg vs. EG: 28.0 ± 9.72 mmHg, *p* < 0.001). Regarding time-domain parameters, SDNN was significantly lower in the EG group than in the PG group (PG: 35.3 ± 20.1 vs. EG: 32.0 ± 18.5, *p* = 0.047). Among the frequency-domain parameters, the EG group showed significantly lower values than the PG group for LnVLF (PG: 5.65 ± 0.75 vs. EG: 5.48 ± 0.69, *p* = 0.006), LnLF (PG: 4.16 ± 1.35 vs. EG: 3.78 ± 1.52, *p* = 0.001), and LnHF (PG: 4.53 ± 1.36 vs. EG: 4.26 ± 1.47, *p* = 0.022). In terms of the APG parameters, the c peak and d peak were significantly lower in the EG group for c peak (PG: −152.9 ± 110.1 vs. EG: −183.1 ± 101.0, *p* = 0.045) and d peak (PG: −322.7 ± 127.6 vs. EG: −360.5 ± 103.5, *p* = 0.026).

**Figure 1:**
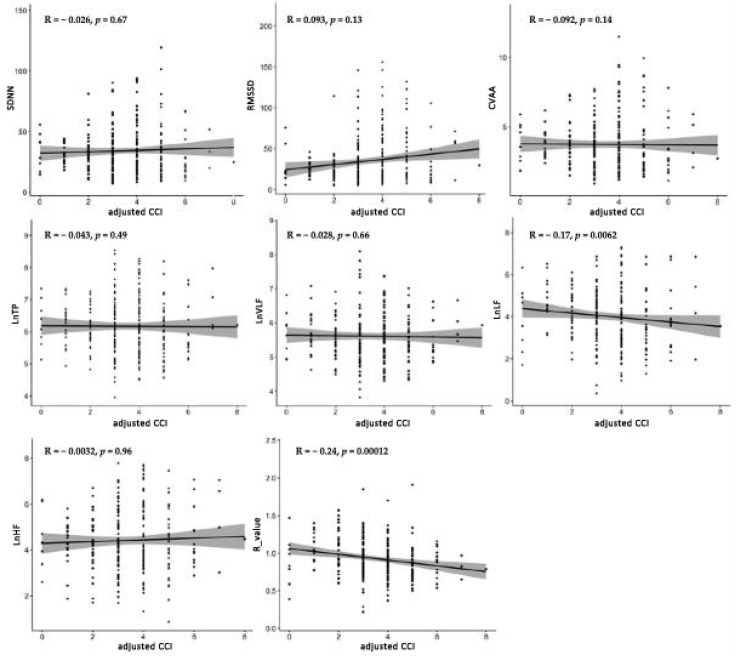
Correlations between Age-Adjusted Charlson Comorbidity Index (ACCI) and Heart Rate Variability (HRV) parameters. Spearman’s correlation coefficients (R) and *p*-values are shown for each parameter. Shaded areas represent 95% confidence intervals. HRV, heart rate variability; ACCI, Age-Adjusted Charlson Comorbidity Index; SDNN, the standard deviation of normal-to-normal intervals; RMSSD, the square root of the mean of the sum of the squared differences between adjacent normal-to-normal intervals; CVRR, the coefficient of variation in R-R intervals; LnTP, natural logarithm Total Power; LnVLF, natural logarithm Very Low Frequency Power; LnLF, natural logarithm Low Frequency; LnHF, natural logarithm High Frequency.

**Figure 2:**
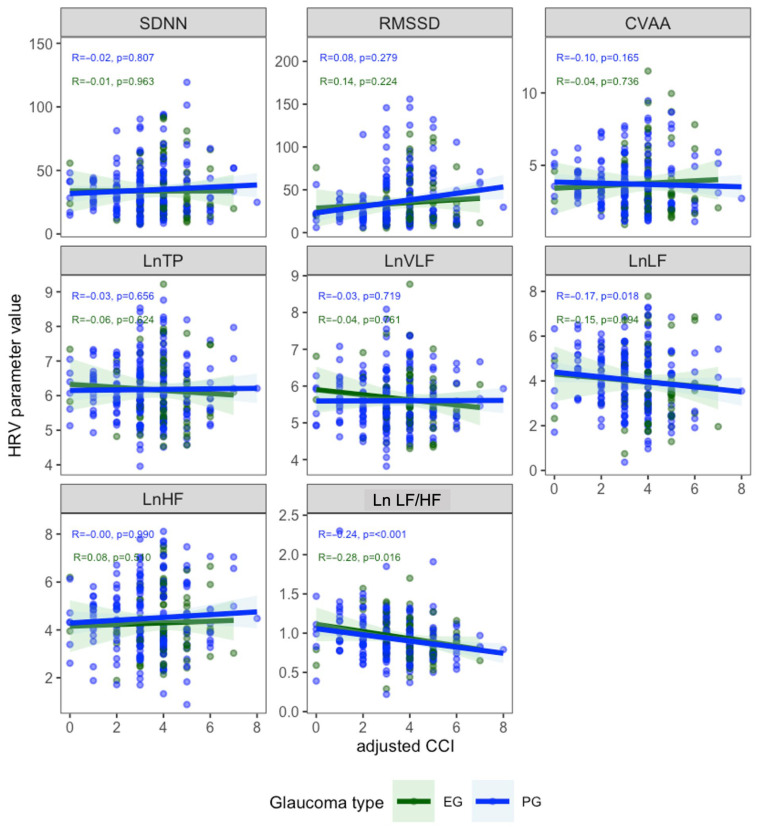
Correlations between Age-Adjusted Charlson Comorbidity Index (ACCI) and Heart Rate Variability (HRV) parameters by glaucoma type. Spearman’s correlation coefficients (R) and *p*-values are shown for each parameter in patients with primary open-angle glaucoma (PG, blue) and exfoliation glaucoma (EG, green). HRV, heart rate variability; ACCI, Age-Adjusted Charlson Comorbidity Index; SDNN, the standard deviation of normal-to-normal intervals; RMSSD, the square root of the mean of the sum of the squared differences between adjacent normal-to-normal intervals; CVRR, the coefficient of variation in R-R intervals; LnTP, natural logarithm Total Power; LnVLF, natural logarithm Very Low Frequency Power; LnLF, natural logarithm Low Frequency; LnHF, natural logarithm High Frequency.

**Figure 3:**
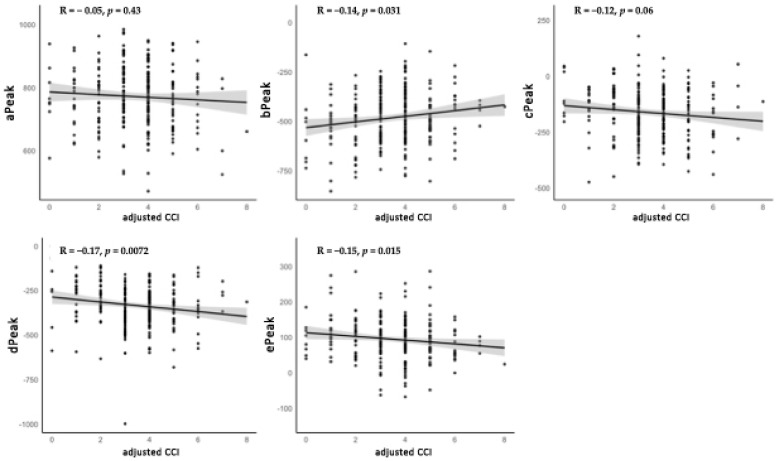
Correlations between Age-Adjusted Charlson Comorbidity Index (ACCI) and Accelerated Plethysmography (APG) parameters. Spearman’s correlation coefficients (R) and *p*-values are shown for each parameter. Shaded areas represent 95% confidence intervals. APG, Accelerated plethysmography; ACCI, Age-Adjusted Charlson Comorbidity Index.

**Figure 4:**
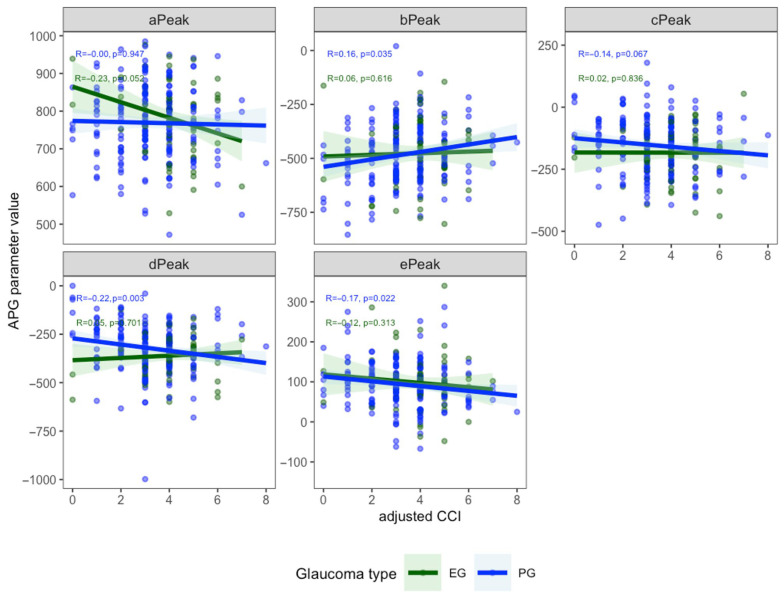
Correlations between Age-Adjusted Charlson Comorbidity Index (ACCI) and Accelerated Plethysmography (APG) parameters by glaucoma type. Spearman’s correlation coefficients (R) and *p*-values are shown for each parameter in patients with primary open-angle glaucoma (PG, blue) and exfoliation glaucoma (EG, green). APG, Accelerated plethysmography; ACCI, Age-Adjusted Charlson Comorbidity Index.

Figure 1 shows the correlations between ACCI and HRV parameters. Significant negative correlations were observed for LnLF (R = −0.17, *p* = 0.0062) and LnLF/LnHF (R = −0.24, *p* = 0.00012), while no significant associations were found for the other parameters. In patients with PG, ACCI was inversely correlated with LnLF (R = −0.17, *p* = 0.018) and LnLF/LnHF (R = −0.24, *p* < 0.001). In patients with EG, ACCI showed a significant negative correlation with LnLF/LnHF (R = −0.28, *p* = 0.016) (Figure 2). Figure 3 demonstrates the correlations between ACCI and APG parameters. Significant negative correlations were observed for d peak (R = −0.17, *p* = 0.0072) and e peak (R = −0.15, *p* = 0.015). Additionally, a significant positive correlation was found for b peak (R = 0.14, *p* = 0.031). As shown in Figure 4, ACCI showed significant inverse correlations with bPeak (R = −0.16, *p* = 0.035), dPeak (R = −0.22, *p* = 0.003), and ePeak (R = −0.17, *p* = 0.022) in patient with PG.

In Table 2, multivariable linear regression analysis revealed a significant positive association between ACCI and RMSSD (coefficient: 2.860; 95% CI: 0.130 to 5.589). In addition, pulse rate was significantly negatively associated with all three time-domain parameters. Table 3 shows the results of the multivariable linear regression analysis for HRV frequency-domain parameters. ACCI was significantly negatively associated with LnLF (coefficient: −0.147; 95% CI: −0.274 to −0.019) and LnLF/LnHF (coefficient: −0.037; 95% CI: −0.062 to −0.011). In addition, pulse rate was significantly negatively associated with all frequency-domain parameters.

Table 4 shows the results of the multivariable linear regression analysis for APG parameters. ACCI was significantly negatively associated with e peak (coefficient: −5.89; 95% CI: −11.7 to −0.120). BMI was significantly associated with a peak and b peak. Pulse rate was significantly associated with a peak, b peak, and c peak. With regard to sBP, the significant associations with b peak and d peak were observed. Additionally, there was a significant association between MD and d peak. Table 5 shows the results of the multivariable logistic regression analysis for vascular types. ACCI was significantly associated with Type B (coefficient: 0.305; 95% CI: 0.057 to 0.552). Type C were significantly associated with pulse rate and sBP.

Table 6 shows the association between HRV and APG parameters and ACCI, stratified by glaucoma type. In the PG group, ACCI was significantly positively associated with RMSSD (coefficient = 3.734, 95% CI: 0.505 to 6.963) and significantly negatively associated with LnLF (coefficient = −0.148, 95% CI: −0.286 to −0.009) and LF/HF (coefficient = −0.035, 95% CI: −0.066 to −0.005). In terms of the APG parameters, c peak was associated with ACCI in the PG group (coefficient = −16.3, 95% CI: −29.2 to −3.42), whereas a peak was associated with ACCI in the EG group (coefficient = −27.7, 95% CI: −45.1 to −10.4).

## 4. Discussion

This cross-sectional study examined the association between ACCI and HRV/APG parameters. We found a significant positive association between ACCI and RMSSD and significant negative associations between ACCI and both LnLF and LnLF/LnHF. Additionally, the negative association between ACCI and e peak was observed in this study. These findings suggest that systemic comorbidity burden may be associated with ANS activity and hemodynamics in patients with glaucoma.

This study suggests that ANS function was lower in patients with advancing age and increasing comorbidity burden. As shown in Table 2 and Table 3, parameters reflecting the balance between sympathetic and parasympathetic activity, such as LnLF and LnLF/LnHF, significantly decreased, whereas parasympathetic parameters, including RMSSD, significantly increased. In several previous studies, a similar trend was observed with increasing age, supporting the present findings [11,33,34,35,36]. Moreover, a previous study of elderly inpatients reported that higher CCI scores were associated with reduced HRV [37], suggesting a link between comorbidity burden and impaired ANS function. These findings are consistent with our results and imply that similar mechanisms may operate in patients with glaucoma.

Several hypotheses can be proposed to explain these findings. First, in glaucoma patients with advanced age and a higher burden of comorbidities, sympathetic activity may be markedly reduced, resulting in a state of relative parasympathetic predominance. Second, in elderly patients with multiple comorbidities, the use of antihypertensive agents or β-blockers may suppress sympathetic activity and enhance parasympathetic dominance. A previous study has reported that β-blocker use is associated with increased RMSSD and HF, as well as decreased LF/HF [38]. Furthermore, Zaliunas et al. demonstrated that during amlodipine use, HF were maintained while LF/HF decreased, indicating a shift toward parasympathetic predominance [39]. Given these findings, medication use may influence autonomic function. However, in this study, medication use was not accounted for as a confounding factor; therefore, future studies should evaluate the impact of medications on autonomic function.

There may be differences in ANS function depending on the type of glaucoma [8,10,11,40,41]. In our previous studies, the EG group exhibited greater sympathetic predominance compared to the PG group [11,18]. In the present study, a significant positive association between ACCI and RMSSD and a significant negative association between ACCI and LnLF and LF/HF were observed in the PG group, whereas these associations were not significant in the EG group. As shown in Table 1, the PG group consisted of younger patients with a significantly lower systemic comorbidity burden compared to the EG group (Age: 65.6 ± 12.7 vs. 74.9 ± 9.45 years, *p* < 0.001; ACCI: 3.25 ± 1.51 vs. 4.00 ± 1.21, *p* < 0.001). A systematic review demonstrated that frail older adults exhibit reduced adaptability of heart rate dynamics compared to their non-frail counterparts [42]. Given their younger age and lower ACCI, the PG group may have retained relatively preserved adaptability of ANS, making the influence of systemic comorbidities on ANS function more apparent in this group. In contrast, in the EG group, impaired tissue integrity and vascular sclerosis may have diminished ANS responsiveness, making the effect of ACCI on HRV less apparent. Exfoliation syndrome (XFS), which underpins EG, is characterized by vascular dysregulations, including endothelial dysfunction [43], oxidative stress [44,45], and abnormalities of coagulation [46]. Additionally, a recent study has shown that systemic arteriosclerosis is more pronounced in patients with EG than in those with PG [23]. These vascular changes may indirectly contribute to dysregulation of ANS. Our findings suggest that ANS dysfunction in PG may primarily result from systemic comorbidities, whereas in EG it may arise from disease-specific mechanisms. Future studies should further evaluate ANS adaptability according to glaucoma subtype.

Furthermore, this study suggests systemic arterial stiffness and atherosclerosis may be associated with both aging and increasing comorbidity burden in patients with glaucoma. As shown in Figure 2, significant correlations were observed between ACCI and several parameters, including the b, d, and e peaks. After adjusting for confounding factors, there was a significant association between ACCI and the e peak, as presented in Table 4. In addition, Table 5 represents that ACCI was significantly associated with Type B. Our previous study demonstrated that all APG components, except for the a peak, were significantly associated with age [23]. Several studies reported that arterial stiffness and atherosclerosis were significantly correlated with higher CCI scores [47,48,49]. Therefore, the findings of our study are consistent with those of previous studies. One possible explanation for the present findings involves the following pathophysiological mechanism. Systemic arterial stiffness and atherosclerosis represent a shared pathophysiological basis among several conditions represented in the ACCI. Arterial wall stiffening associated with aging may increase the risk of developing multiple vascular-related comorbidities. Conversely, the presence of various chronic comorbidities may promote the progression of arterial stiffening through tissue remodeling processes. This study provides new insights into the relationship between arterial stiffness and comorbidity burden in glaucoma patients. Future studies are needed to further examine these relationships in patients with glaucoma.

There may be differences in the relationship between arterial stiffness and comorbidity burden among glaucoma subtypes. In this study, a significant association between a peak and ACCI was observed in the EG group. Our previous study demonstrated a significant association between a peak and pseudoexfoliation material (PEM) [23]. In EG, the accumulation of PEM in vascular walls may impair arterial compliance, leading to reduced early systolic acceleration as reflected by a lower a peak. This vascular dysfunction may underlie the observed association between a peak and comorbidity burden in patients with EG. Additionally, a significant association between c Peak and ACCI was observed only in PG in this study. patients with PG may retain relatively preserved vascular function, allowing c Peak to more sensitively reflect systemic vascular burden. Further studies are needed to clarify the relationship between ACCI and APG parameters in each glaucoma subtype.

Visual field impairment may be related to arterial stiffness. As shown in Table 4, there was a significant association between MD and d peak in glaucoma patients (coefficient = 2.66, 95% CI: 0.160 to 5.16). Several studies reported that systemic arterial stiffness and atherosclerosis are linked to glaucoma progression and visual field loss [50,51]. These associations may be influenced by various factors, including reduced blood flow to the optic nerve [4,7] and oxidative stress [44].

This study has several limitations. First, this cross-sectional study was unable to clarify the causal relationship between ACCI and HRV/APG changes. Second, the timing of HRV and APG measurement was not controlled which may have impacted the results. Third, medications with potential effects on autonomic and vascular function (e.g., β-blockers, calcium channel blockers, ACE inhibitors) were not considered as confounders as mentioned in the Discussion. Future studies should include detailed information on systemic and topical medications. Fourth, no a priori sample size calculation was performed, because no prior studies were available to estimate the expected effect size. In this study, all consecutive eligible patients during the study period were included. Fifth, autonomic and vascular changes in glaucoma may be affected by the timing of measurements. Future studies will need to take the timing of measurements into account. Sixth, this single-center study conducted in a university hospital may limit the generalizability of the findings.

## 5. Conclusions

This study investigated the association between ACCI and HRV and APG parameters. Our findings suggest that a higher systemic comorbidity burden may be associated with impaired ANS activity and hemodynamic regulation in patients with glaucoma. Further studies are needed to elucidate the underlying mechanisms.

## Figures and Tables

**Table 1:** Comparison of demographic data and Heart Rate Variability (HRV) and Accelerated Plethysmography (APG) parameters between open-angle glaucoma (PG) and exfoliation glaucoma (EG) groups.

Parameters	PG		EG		*p*-Value ^a^	Total	
	N or Mean	% or SD	N or Mean	% or SD		N or Mean	% or SD
Subjects	186		74			260	
Eyes	186		74			260	
Age, years	66.3	12.2	74.8	8.53	<0.001	68.8	11.9
Sex, Female	87	46.8	33	44.6	0.857	120	46.2
BMI, kg/m^2^	22.8	3.67	22.7	3.50	0.805	22.7	3.62
sBP, mmHg	141.2	14.0	149.5	22.8	0.007	143.6	21.9
dBP, mmHg	81.0	13.1	83.7	16.2	0.193	81.8	14.7
Pulse rate, bpm	73.1	13.2	76.0	14.6	0.130	73.9	13.6
IOP (highest recorded), mmHg	20.3	7.39	28.0	9.72	<0.001	22.5	8.81
Medication score, points	2.67	1.63	3.03	1.93	0.165	2.77	1.72
MD, dB	−6.06	6.40	−6.23	6.96	0.861	−6.10	6.54
ACCI, points	3.25	1.51	4.00	1.21	<0.001	3.46	1.47
Time-domain parameters							
SDNN	35.3	20.1	32.0	18.5	0.047	34.3	20.0
RMSSD	35.1	28.5	34.7	25.5	0.844	35.3	29.3
CVAA	3.86	1.87	3.57	2.02	0.072	3.73	1.86
Frequency-domain parameters							
LnTP	6.24	0.89	6.04	0.90	0.010	6.17	0.90
LnVLF	5.65	0.75	5.48	0.69	0.006	5.61	0.74
LnLF	4.16	1.35	3.78	1.52	0.001	4.01	1.38
LnHF	4.53	1.36	4.26	1.47	0.022	4.43	1.41
LnLF/LnHF	0.95	0.29	0.92	0.32	0.170	0.93	0.27
Accelerated plethysmography							
a Peak	769.0	98.2	782.4	89.1	0.318	772.8	95.7
b Peak	−483.8	136.6	−476.2	144.6	0.697	−481.6	138.6
c Peak	−152.9	110.1	−183.1	101.0	0.045	−161.5	108.2
d Peak	−322.7	127.6	−360.5	103.5	0.026	−333.4	122.2
e Peak	94.0	56.7	97.3	65.3	0.690	94.9	59.2
Vascular type							
Unknown	5	2.7	2	2.7	0.440	7	2.7
Type A	55	29.6	15	20.3		70	26.9
Type B	104	55.9	45	60.8		149	57.3
Type C	22	11.8	12	16.2		34	13.1

^a^*p* values are calculated using the unpaired t-test or chi-square test. PG, Primary open-angle Glaucoma; EG, Exfoliation Glaucoma; CCI, Charlson Comorbidity Index; ACCI, Age-Adjusted Charlson Comorbidity Index; BMI, body mass index; sBP, systolic blood pressure; dBP, diastolic blood pressure; bpm, beats per minute; SD, standard deviation; IOP, intraocular pressure; MD, mean deviation; SDNN, the standard deviation of normal-to-normal intervals; RMSSD, the square root of the mean of the sum of the squared differences between adjacent normal-to-normal intervals; CVRR, the coefficient of variation in R-R intervals; LnTP, natural logarithm Total Power; LnVLF, natural logarithm Very Low Frequency Power; LnLF, natural logarithm Low Frequency; LnHF, natural logarithm High Frequency.

**Table 2:** Multivariable linear regression analysis of Age-Adjusted Charlson Comorbidity Index (ACCI) and time-domain Heart Rate Variability (HRV) parameters.

	HRV Time Domain Parameters (Coefficient [95% CI])		
Variables	SDNN	RMSSD	CVAA
ACCI, points	0.152	2.860 *	−0.053
	[−1.718 to 2.022]	[0.130 to 5.589]	[−0.236 to 0.131]
Sex, F/M	0.911	3.673	0.059
	[−4.226 to 6.048]	[−3.824 to 11.17]	[−0.445 to 0.563]
BMI, kg/m^2^	−0.125	−0.270	−0.008
	[−0.851 to 0.599]	[−1.328 to 0.789]	[−0.079 to 0.063]
Pulse Rate, /bpm	−0.657 ***	−0.956 ***	−0.032 ***
	[−0.849 to −0.466]	[−1.235 to −0.677]	[−0.051 to −0.013]
sBP, /mmHg	−0.084	−0.099	−0.012
	[−0.257 to 0.089]	[−0.352 to 0.153]	[−0.028 to 0.005]
dBP, /mmHg	0.215	0.201	0.019
	[−0.045 to 0.474]	[−0.178 to 0.580]	[−0.006 to 0.045]
IOP (highest recorded), mmHg	−0.051	−0.135	−0.006
	[−0.377 to 0.274]	[−0.610 to 0.340]	[−0.038 to 0.026]
Medication score, points	0.149	0.317	0.036
	[−1.317 to 1.616]	[−0.182 to 2.457]	[−0.107 to 0.180]
MD, dB	0.213	0.328	0.022
	[−0.177 to 0.603]	[−0.242 to 0.897]	[−0.016 to 0.061]
Glaucoma type, PG/EG	−0.449	−0.219	−0.127
	[−6.695 to 5.797]	[−9.336 to 8.897]	[−0.740 to 0.485]
Adjusted R^2^	0.160	0.186	0.030

*** *p* < 0.001, * *p* < 0.05. Adjusted for sex, BMI, pulse rate, systolic and diastolic BP, IOP (highest recorded), medication score, MD, and glaucoma type. HRV, heart rate variability; CI, confidence interval; SDNN, the standard deviation of normal-to-normal intervals; RMSSD, the square root of the mean of the sum of the squared differences between adjacent normal-to-normal intervals; CVRR, the coefficient of variation in R-R intervals; ACCI, Age-Adjusted Charlson Comorbidity Index; BMI, body mass index; sBP, systolic blood pressure; dBP, diastolic blood pressure; bpm, beats per minute.

**Table 3:** Multivariable linear regression analysis of Age-Adjusted Charlson Comorbidity Index (ACCI) and frequency-domain Heart Rate Variability (HRV) parameters.

	HRV Frequency Domain Parameters (Coefficient [95% CI])				
Variables	LnTP	LnVLF	LnLF	LnHF	LnLF/LnHF
ACCI, points	−0.048	−0.061	−0.147 *	−0.001	−0.037 **
	[−0.127 to 0.031]	[−0.126 to 0.005]	[−0.274 to −0.019]	[−0.129 to 0.127]	[−0.062 to −0.011]
Sex, F/M	0.076	0.106	0.030	0.038	0.013
	[−0.141 to 0.293]	[−0.075 to 0.286]	[−0.320 to 0.380]	[−0.313 to 0.389]	[−0.058 to 0.083]
BMI, kg/m^2^	−0.012	−0.022	−0.013	−0.003	−0.004
	[−0.043 to 0.018]	[−0.048 to 0.003]	[−0.062 to 0.037]	[−0.053 to 0.047]	[−0.014 to 0.006]
Pulse Rate, /bpm	−0.034 ***	−0.025 ***	−0.033 ***	−0.052 ***	0.004 **
	[−0.042 to −0.026]	[−0.031 to −0.018]	[−0.046 to −0.020]	[−0.065 to −0.039]	[0.001 to 0.007]
sBP, /mmHg	−0.002	−0.001	−0.002	−0.003	0.001
	[−0.009 to 0.006]	[−0.007 to 0.005]	[−0.014 to 0.010]	[−0.015 to 0.009]	[−0.002 to 0.002]
dBP, /mmHg	0.007	0.003	0.014	0.008	0.002
	[−0.014 to 0.014]	[−0.006 to 0.012]	[−0.004 to 0.031]	[−0.010 to 0.026]	[−0.001 to 0.006]
IOP (highest recorded), mmHg	0.005	0.003	−0.007	0.001	−0.002
	[−0.051 to 0.073]	[−0.008 to 0.015]	[−0.029 to 0.016]	[−0.021 to 0.023]	[−0.006 to 0.002]
Medication score, points	0.011	0.013	0.010	−0.011	0.007
	[−0.004 to 0.017]	[−0.038 to 0.065]	[−0.090 to 0.110]	[−0.112 to 0.089]	[−0.013 to 0.028]
MD, dB	0.009	0.001	0.023	0.016	0.002
	[−0.007 to 0.026]	[−0.013 to 0.014]	[−0.004 to 0.050]	[−0.010 to 0.043]	[−0.004 to 0.007]
Glaucoma type, PG/EG	−0.072	−0.111	0.025	0.083	0.015
	[−0.335 to 0.192]	[−0.331 to 0.108]	[−0.401 to 0.451]	[−0.344 to 0.510]	[−0.071 to 0.101]
Adjusted R^2^	0.232	0.199	0.116	0.205	0.077

*** *p* < 0.001, ** *p* < 0.01, * *p* < 0.05. Adjusted for sex, BMI, pulse rate, systolic and diastolic BP, IOP (highest recorded), medication score, MD, and glaucoma type. HRV, heart rate variability; CI, confidence interval; LnTP, natural logarithm Total Power; LnVLF, natural logarithm Very Low Frequency Power; LnLF, natural logarithm Low Frequency; LnHF, natural logarithm High Frequency; ACCI, Age-Adjusted Charlson Comorbidity Index; BMI, body mass index; sBP, systolic blood pressure; dBP, diastolic blood pressure; bpm, beats per minute; IOP, intraocular pressure; MD, mean deviation.

**Table 4:** Multivariable linear regression analysis of Age-Adjusted Charlson Comorbidity Index (ACCI) and Accelerated Plethysmography (APG) parameters.

	APG Parameters (Coefficient [95% CI])				
Variables	a Peak	b Peak	c Peak	d Peak	e Peak
ACCI, points	−5.23	8.04	−9.72	−4.66	−5.89 *
	[−12.7 to 4.51]	[−2.91 to 21.0]	[−20.3 to 0.860]	[−16.8 to 7.44]	[−11.7 to −0.120]
Sex, F/M	3.09	−29.9	10.0	9.68	4.91
	[−20.8 to 29.7]	[−68.0 to 2.10]	[−19.6 to 39.6]	[−24.2 to 43.5]	[−11.2 to 21.0]
BMI, kg/m^2^	4.76 *	−6.84 **	−2.43	0.020	1.50
	[1.82 to 8.60]	[−11.9 to −2.45]	[−6.51 to 1.65]	[−4.65 to 4.69]	[−0.727 to 3.72]
Pulse Rate, /bpm	0.975	−2.40 **	−1.85 **	1.40 *	0.543
	[−0.038 to 1.82]	[−4.08 to −1.49]	[−2.99 to −0.719]	[0.101 to 2.70]	[0.076 to 1.16]
sBP, /mmHg	0.326	1.62 *	0.449	−1.41 *	−0.265
	[−0.475 to 1.22]	[0.277 to 2.63]	[−0.554 to 1.45]	[−2.56 to −0.261]	[−0.812 to 0.282]
dBP, /mmHg	−0.988	0.241	0.417	0.336	0.056
	[−2.47 to 0.162]	[−1.40 to 2.26]	[−1.13 to 1.96]	[−1.43 to 2.11]	[−0.788 to 0.899]
IOP (highest recorded), mmHg	−0.078	−0.368	1.42	1.32	0.131
	[−1.810 to 1.653]	[−2.72 to 1.98]	[−0.455 to 3.291]	[−0.825 to 3.46]	[−0.889 to 1.15]
Medication score, points	1.860	−3.59	−2.98	1.32	3.00
	[−5.911 to 9.631]	[−14.1 to 6.95]	[−11.4 to 5.43]	[−8.30 to 10.9]	[−1.58 to 7.58]
MD, dB	−1.191	−1.64	1.75	2.66 *	−0.155
	[−3.210 to 0.829]	[−4.38 to 1.10]	[−0.430 to 3.29]	[0.160 to 5.16]	[−1.35 to 1.04]
Glaucoma type, PG/EG	−17.72	10.41	31.1	34.7	−3.27
	[−50.18 to 14.75]	[−33.6 to 54.4]	[−3.97 to 66.3]	[−5.52 to 74.8]	[−22.4 to 15.9]
Adjusted R^2^	0.025	0.110	0.083	0.071	0.022

** *p* < 0.01, * *p* < 0.05. Adjusted for sex, BMI, pulse rate, systolic and diastolic BP, IOP (highest recorded), medication score, MD, and glaucoma type. APG, Accelerated plethysmography; CI, confidence interval; ACCI, Age-Adjusted Charlson Comorbidity Index; BMI, body mass index; sBP, systolic blood pressure; dBP, diastolic blood pressure; bpm, beats per minute; IOP, intraocular pressure; MD, mean deviation.

**Table 5:** Multivariable logistic regression analysis of Age-Adjusted Charlson Comorbidity Index (ACCI) and vascular type.

	APG Parameters (Coefficient [95% CI])	
Variables	Type B (vs. Type A)	Type C (vs. Type A)
ACCI, points	0.305 *	0.099
	[0.057 to 0.552]	[−0.290 to 0.488]
Sex, F/M	−0.443	−1.01
	[−1.12 to 0.231]	[−2.04 to 0.020]
BMI, kg/m2	−0.047	−0.063
	[−0.138 to 0.044]	[−0.219 to 0.093]
Pulse Rate, /bpm	−0.007	−0.061 **
	[−0.032 to 0.018]	[−0.106 to −0.016]
sBP, /mmHg	0.000	0.038 *
	[−0.024 to 0.024]	[0.006 to 0.069]
dBP, /mmHg	0.024	0.010
	[−0.013 to 0.061]	[−0.039 to 0.059]
IOP (highest recorded), mmHg	−0.007	−0.030
	[−0.048 to 0.034]	[−0.100 to 0.040]
Medication score, points	−0.105	−0.065
	[−0.297 to 0.086]	[−0.349 to 0.220]
MD, dB	−0.038	−0.057
	[−0.092 to 0.016]	[−0.133 to 0.019]
Glaucoma type, PG/EG	−0.134	0.343
	[−0.978 to 0.709]	[−1.544 to 0.858]

** *p* < 0.01, * *p* < 0.05. Adjusted for sex, BMI, pulse rate, systolic and diastolic BP, IOP (highest recorded), medication score, MD, and glaucoma type. APG, Accelerated plethysmography; CI, confidence interval; ACCI, Age-Adjusted Charlson Comorbidity Index; BMI, body mass index; sBP, systolic blood pressure; dBP, diastolic blood pressure; bpm, beats per minute; IOP, intraocular pressure; MD, mean deviation.

**Table 6:** Association between Age-Adjusted Charlson Comorbidity Index (ACCI) and Heart Rate Variability (HRV) and Accelerated Plethysmography (APG) parameters stratified by glaucoma type.

HRV Parameters	PG (Coefficient [95% CI])	EG (Coefficient [95% CI])
Time-domain parameters		
SDNN	0.435 [−1.679 to 2.550]	−0.667 [−5.327 to 3.993]
RMSSD	3.734 [0.505 to 6.963] *	0.381 [−5.744 to 6.505]
CVRR	−0.060 [−0.255 to 0.135]	−0.005 [−0.523 to 0.512]
Frequency-domain parameters		
LnTP	−0.045 [−0.134 to 0.044]	−0.048 [−0.245 to 0.149]
LnVLF	−0.059 [−0.132 to 0.014]	−0.073 [−0.243 to 0.097]
LnLF	−0.148 [−0.286 to −0.009] *	−0.085 [−0.428 to 0.257]
LnHF	0.010 [−0.142 to 0.162]	−0.018 [−0.305 to 0.270]
LnLF/LnHF	−0.035 [−0.066 to −0.005] *	−0.030 [−0.089 to 0.029]
Accelerated plethysmography		
a Peak	1.273 [−10.841 to 13.387]	−27.722 [−45.086 to −10.357] **
b Peak	6.179 [−9.570 to 21.929]	10.961 [−17.724 to 39.647]
c Peak	−16.308 [−29.194 to −3.422] *	7.211 [−13.406 to 27.828]
d Peak	−6.610 [−21.448 to 8.228]	7.874 [−14.863 to 30.611]
e Peak	−6.104 [−12.905 to 0.697]	−7.621 [−20.850 to 5.608]
Type B (vs. Type A)	0.290 [−0.009 to 0.590]	0.905 [−0.024 to 1.834]
Type C (vs. Type A)	−0.119 [−0.638 to 0.401]	5.130 [−1.016e6 to 1.017e6]

** *p* < 0.01, * *p* < 0.05. Adjusted for sex, BMI, pulse rate, systolic and diastolic BP, IOP (highest recorded), medication score, and MD. HRV, heart rate variability; CI, confidence interval; SDNN, the standard deviation of normal-to-normal intervals; RMSSD, the square root of the mean of the sum of the squared differences between adjacent normal-to-normal intervals; CVRR, the coefficient of variation in R-R intervals; LnTP, natural logarithm Total Power; LnVLF, natural logarithm Very Low Frequency Power; LnLF, natural logarithm Low Frequency; LnHF, natural logarithm High Frequency; ACCI, Age-Adjusted Charlson Comorbidity Index; IOP, intraocular pressure; MD, mean deviation.

## Data Availability

Data is fully available upon reasonable request to corresponding authors.

## Abbreviations

The following abbreviations are used in this manuscript:

HRV	Heart rate variability
APG	Acceleration plethysmography
ANS	Autonomic nervous system
CCI	Charlson Comorbidity Index
ACCI	Age-adjusted Charlson Comorbidity Index
SDNN	The standard deviation of normal-to-normal intervals
RMSSD	The square root of the mean of the sum of the squared differences between adjacent normal-to-normal intervals
CVRR	The coefficient of variation in R-R intervals
TP	Total power
VLF	Very-low-frequency
LF	Low-frequency
HF	High-frequency
PG	Primary open-angle glaucoma
EG	Exfoliation glaucoma
IOP	Intraocular pressure
OPP	Ocular perfusion pressure
BMI	Body mass index
BP	Blood pressure
CI	Confidence interval
XFS	Exfoliation syndrome
PEM	Pseudoexfoliation material
MD	Mean deviation
